# Doxycycline Promotes Carcinogenesis & Metastasis via Chronic Inflammatory Pathway: An *In Vivo* Approach

**DOI:** 10.1371/journal.pone.0151539

**Published:** 2016-03-21

**Authors:** Neha Nanda, Devinder K. Dhawan, Alka Bhatia, Akhtar Mahmood, Safrun Mahmood

**Affiliations:** 1 Department of Experimental Medicine and Biotechnology, Postgraduate Institute of Medical Education and Research (PGIMER), Chandigarh, India; 2 Department of Biophysics, Panjab University, Chandigarh, India; 3 Department of Biochemistry, Panjab University, Chandigarh, India; Yong Loo Lin School of Medicine, National University of Singapore, SINGAPORE

## Abstract

**Background:**

Doxycycline (DOX) exhibits anti-inflammatory, anti-tumor, and pro-apoptotic activity and is being tested in clinical trials as a chemotherapeutic agent for several cancers, including colon cancer.

**Materials & Methods:**

In the current study, the chemotherapeutic activity of doxycycline was tested in a rat model of colon carcinogenesis, induced by colon specific cancer promoter, 1,2, dimethylhydrazine (DMH) as well as study the effect of DOX-alone on a separate group of rats.

**Results:**

Doxycycline administration in DMH-treated rats (DMH-DOX) unexpectedly increased tumor multiplicity, stimulated progression of colonic tumor growth from adenomas to carcinomas and revealed metastasis in small intestine as determined by macroscopic and histopathological analysis. DOX-alone treatment showed markedly enhanced chronic inflammation and reactive hyperplasia, which was dependent upon the dose of doxycycline administered. Moreover, immunohistochemical analysis revealed evidence of inflammation and anti-apoptotic action of DOX by deregulation of various biomarkers.

**Conclusion:**

These results suggest that doxycycline caused chronic inflammation in colon, small intestine injury, enhanced the efficacy of DMH in tumor progression and provided a mechanistic link between doxycycline-induced chronic inflammation and tumorigenesis. Ongoing studies thus may need to focus on the molecular mechanisms of doxycycline action, which lead to its inflammatory and tumorigenic effects.

## Introduction

Colon cancer is the third most common cancer worldwide, which is responsible for about 10% of total cancer related mortality [1]. Despite decades of research and the current development in cancer therapy, the 5-year survival rate for colon cancer at distant stage is about 13% [1,2]. Thus, the present scenario of the colon cancer indicates that alternative approaches to control this malignant neoplastic disease, are critically required.

Doxycycline (DOX), an analogue of tetracycline derived from oxytetracycline or methacycline, has been widely used in the treatment of several infectious diseases with tolerable side effects [3]. Doxycycline, a potent non-specific inhibitor of MMPs, exhibits cytotoxic, anti-inflammatory, anti-invasive, anti-proliferative, anti-angiogenic, and pro-apoptotic properties in a variety of cancers [4–6]. A number of *in vitro* studies using colon cancer cell lines have revealed that doxycycline exerts potent chemotherapeutic activity, alone and in combination with other therapeutic compounds [7,8]. Also, it has been reported that doxycycline administration decreases the expression and transcriptional activity of certain oncogenes in colorectal cancer [9]. However, additional investigations are required to evaluate the role of doxycycline and its underlying mechanisms in this disease, especially under *in vivo* conditions.

Animal models of colon carcinogenesis provide tools for appraisal of the efficacy and toxicity of chemotherapeutic agents, and also allow for the evaluation of various biomarkers, that can be used as clinical endpoints for therapeutic interventions and gain insight into mechanisms whereby novel agents may act, or as predictions of anticancer activity. Therefore, the present study was initiated to determine the potential chemotherapeutic activity of doxycycline in rats subjected to DMH induced colon carcinogenesis. Interestingly, it was observed that treatment with DMH followed by DOX unexpectedly promoted the colon cancer progression and metastasis, thereby indicating that DOX treatment not only failed to suppress DMH induced colonic lesions, but in turn accelerated progression of molecular events leading to colon carcinogenesis. Moreover, normal rats treated with DOX-alone showed increased evidence of chronic inflammation to reactive hyperplasia. We also observed changes in the expression of various biomarker proteins involved in the different stages of colon carcinogenesis to substantiate the carcinogenic potential of doxycycline treatment.

## Materials and Methods

### Ethics statement

All the animal procedures were performed in accordance with ethical guidelines for care and use of laboratory animals of the National Institutes of Health. The study was approved by Institutional Animal Ethics Committee (IAEC), PGIMER vide Reference No. 50/IAEC/247.All efforts were made to minimize suffering, animals received proper veterinary care, and all the animals were survived during the entire course of the study, so euthanization was not required.

### Animals

Healthy male Sprague Dawley rats with body weight ranging from 180 to 200g were divided into four groups of 24 rats in each group. Group I (Normal Controls) received weekly subcutaneous (s.c.) injections of normal saline, while Group II rats were injected with 1, 2 dimethylhydrazine (DMH) (s.c) (Sigma) at a dosage of 30mg/kg body weight [10,11], weekly for 10 weeks (Adenoma) and 20 weeks (Carcinoma). DMH was freshly prepared in normal saline, pH adjusted to 7.0 using dilute NaOH solution. Group III animals were administered DMH for 10 weeks followed by Doxycycline treatment intraperitoneally (i.p) at a dose of 10mg/kg (DMH-DOX (10mg/kg)) or 20mg/kg body weight (DMH-DOX (20mg/kg)) [12,13] per day for 15 days. Animals in Group IV were administered Doxycycline alone (i.p) with different doses comprising 5mg/kg (DOX (5mg/kg)), 10mg/kg (DOX (10mg/kg)) and 20mg/kg body weight (DOX (20mg/kg)) per day for 15 days. Doxycycline (DOX) (Doxycycline hyclate D9891, Lot#BCBD5187V) was purchased from Sigma Aldrich, St. Louis, USA and was freshly prepared in normal saline, pH adjusted to 7.0 using dilute NaOH solution.

### Tumor Analysis

Tumor multiplicity was calculated as number of tumors per group/number of rats per group. Tumor growth was monitored by measuring the tumor length (L) and width (W) using calipers and tumor area was calculated using the formula: A = (L×W). Ellipsoid tumor volume was calculated as length × width^2^× π/6 [14].

### Histology

Tissues from normal as well as tumorous regions were isolated and fixed in 10% paraformaldehyde/PBS solution, embedded in paraffin and sectioned at 4 μm. Paraffin sections were stained with haematoxylin and eosin for routine histological analysis as described by Fischer et al. (2008) [15]. Severity of inflammation was scored on a 0–4 scale (0, normal mucosa; 1, minimal inflammation; 2, mild inflammation; 3, moderate inflammation; and 4, severe inflammation) as described by Wu et al. (2009) [16].

### Immunohistochemistry

Tissues in paraffin block were sectioned at 4μm thickness and were processed for immunostaining. Sections were incubated with primary antibodies diluted (1:100) in 2.5% normal horse serum (NHS; Vector Labs) overnight at 4°C, then stained with biotin-conjugated secondary antibodies followed by incubation with peroxidase-streptavidin complex using Vectastain^®^ Universal Quick Kit (Vector Labs). Immunostaining was performed using 3, 3’-diaminobenzidine (DAB; Sigma) according to the manufacturer’s instructions. Sections were counterstained with haematoxylin for 1 min, mounted, and coverslip was permanently added for light microscopy. IHC scoring was done by using IHC profiler, an open source plugin, as described by Varghese et al. (2014) [17]. The score was assigned in a four tier system (0–3) viz. high positive (3+), positive (2+), low positive (1+) and negative (0).

### Statistical Methods

Statistical analysis between the groups was done by ANOVA. Values with P<0.05 were considered statistically significant.

## Results

### Induction of colon carcinogenesis by DMH and enhancement of its action by Doxycycline treatment

**Fig 1:**
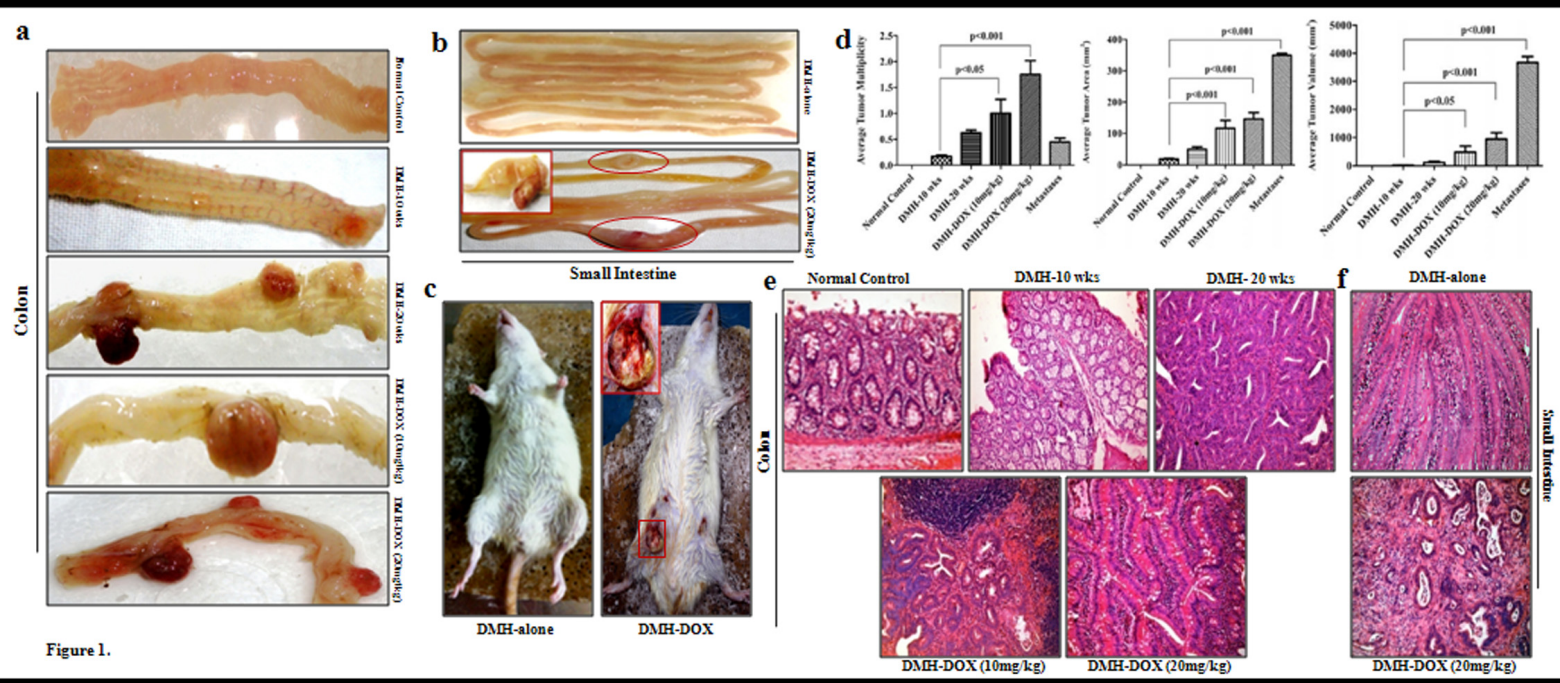
DMH induces colon tumorigenesis and DMH-DOX enhances colon tumor growth and metastasis. **a)** Macroscopic representative samples of normal control, DMH-10 wks, DMH-20 wks, DMH-DOX (10mg/kg), DMH-DOX (20mg/kg) of colon and **b)** DMH-alone, DMH-DOX (20mg/kg) (metastastic) of small intestine **c)** Skin abscesses on injection site of DMH-DOX **d)** Average tumor multiplicity for each treatment group is expressed as number of tumors per rat/total number of rats with tumors ±SE; Average tumor area was calculated as, A = (L×W); and average tumor volume as length × width^2^× π/6 in all treatments **e)** Histopathology of rat colon and **f)** small intestine tissue. Hematoxylin and eosin stain of tissue from control and treated rats (n = 24); Magnification ×200. Data are presented as Mean ± SD.

In the present study, animals injected with DMH usually developed diarrhea after 4–5 weeks, with resolution of the symptoms after 10 and 20 weeks of treatment. A steady increase in the body weights of rats following DMH-10 weeks and DMH-DOX treatments were observed as compared to normal control (Tables 1 and 2). However, a rapid decrease in body weights was observed after 10 weeks till 20 weeks of DMH treatment as compared to control group (Table 3). At 10 weeks, DMH treated rats exhibited a pronounced increase in colonic thickness, vascular congestion, and visible polypoid lesions, whereas multiple tumors were grossly observed at 20 weeks of treatment interval (Fig 1A). Since doxycycline treatment is reported to regress tumor progression in experimental studies, we further evaluated the effectiveness of DOX as a chemotherapeutic agent in DMH induced colon carcinogenesis. Surprisingly, it was noticed that treatment of rats with DMH-DOX (10mg/kg) promoted the colonic tumor growth, whereas DMH-DOX (20mg/kg) treated rats showed a marked increased tumor growth in colons (Fig 1A) as well as rapidly stimulated metastasis in small intestine as compared to DMH-alone treated rats (Fig 1B). Further, all the DMH-DOX treated rats showed visible skin abscesses at injection site (Fig 1C). Due to these unusual skin abscesses at the injection site of DMH-DOX treated rats, we cross-checked these results by switching doxycycline injection from intraperitoneally (i.p.) to subcutaneously (s.c.) injection mode in different group of animals. This switching was done to check, whether these abscesses were due to experimental error, mode of injection or due to the adverse effect of doxycycline. Interestingly, we found same results relating to skin abscesses upon subcutaneous injections of doxycycline treatment in all the animals. Therefore, these findings confirmed that the observed changes were not an experimental error, but were due to doxycycline treatment. Since, DMH was also given subcutaneously, but we failed to find any abscesses in DMH treated rats throughout the study (Fig 1C). With these unusual findings observed in rats treated with DMH for 10 weeks followed by doxycycline treatment for 15 days, these experimental animals could not be continued for 20 weeks of DMH treatment due to substantial damage to colons.

**Table 1:** Effect of DMH treatment for 10 weeks on body weights of rats.

Weeks	Normal Control-10 wks	DMH-10 wks
0	196.8±5.2	198.5±6.45
2	203.12±12.9	234.44±15
4	224.75±21.5	258±13.8
6	228.62±4.06	277.88±7.69^x^^a^
8	240.62±4.41	303.44±7.54^y^^a^
10	250.62±3.71	313.05±1.49^y^^a^

Weight in grams

Data are presented as Mean ± SD

n = 6 for normal control group and n = 12 for treatment group

^x^ p<0.05

^y^ p<0.01

^a^ vs control

**Table 2:** Effect of 10 weeks DMH treatment followed by DOX for 15 days on body weights of rats.

Weeks	Normal Control-12 wks	DMH-DOX (10mg/kg)	DMH-DOX (20mg/kg)
0	198.33±2.51	198.66±3.05	197±2.64
2	208±16.49	187.37±10.6	194.93±8.57
4	233.16±27.57	225.93±17.9	235.68±17
6	236.16±5.89	273.56±6.09	278.93±9.98
8	250.16±7.3	315.43±7.51^y^^a^	318.43±4.5^z^^a^
10	260.83±4	320.43±8.22^y^^a^	328±4.59^y^^a^
12	269.83±3.06	323.18±4.86^x^^a^	343.93±1.14^z^^a^

Weight in grams

Data are presented as Mean ± SD

n = 6 for normal control group and n = 12 for treatment group

^x^ p<0.05

^y^ p<0.01

^z^ p<0.001

^a^ vs control

**Table 3:** Effect of DMH treatment for 20 weeks on body weights of rats.

Weeks	Normal Control-20 wks	DMH-20 wks
0	198.4±3.84	198±6.97
2	199.6±0.84	201±10.32
4	208.4±5.37	227.3±5.09
6	207.7±2.12	242.45±5.44^y^^a^
8	226.1±3.53	253.45±1.48^x^^a^
10	242.3±2.4	248.2±7.77
12	266.3±14.28	260.55±2.47
14	292.4±10.46	262.05±4.17^y^^a^
16	317.9±2.96	266.35±2.61^z^^a^
18	326.1±4.1	290.4±3.25^z^^a^
20	344±5.93	291.55±2.75^z^^a^

Weight in grams

Data are presented as Mean ± SD

n = 6 for normal control group and n = 12 for treatment group

^x^ p<0.05

^y^ p<0.01

^z^ p<0.001

^a^ vs control

Comparing with DMH-alone, treatment with DMH followed by DOX, resulted in a statistically significant increase in tumor multiplicity, as assessed by counting the number of visible surface tumors (Fig 1D). Also, we calculated the tumor growth and observed an increase in average tumor area and tumor volume in all the groups studied (Fig 1D). Histopathology of 10 weeks DMH-treated colons confirmed villous adenoma by an increase in inflammation, hyperplasia and villi-form transformation of the mucosa, whereas adenocarcinoma was confirmed by the presence of dysplastic crypts, nuclear atypia as well as gastrointestinal intraepithelial neoplasia (GIN) foci after 20 weeks treatment as compared to the normal colonic mucosa. Nonetheless, treatment of rats with DMH-DOX (10mg/kg) and DMH-DOX (20mg/kg) showed large lymphoid follicle with the presence of germinal centers, loss of goblet cells and crypts loss as compared to normal colon which conserved their normal mucosal architecture (Fig 1E). Likewise, small intestine of DMH-DOX (20mg/kg) treated rats showed the presence of adenocarcinoma in the course of nuclear expansion, nuclear atypia and lymphoid hyperplasia as compared to DMH-alone treated rats (Fig 1F).

### Induction of chronic inflammation in colon and small intestine by DOX treatment

**Fig 2:**
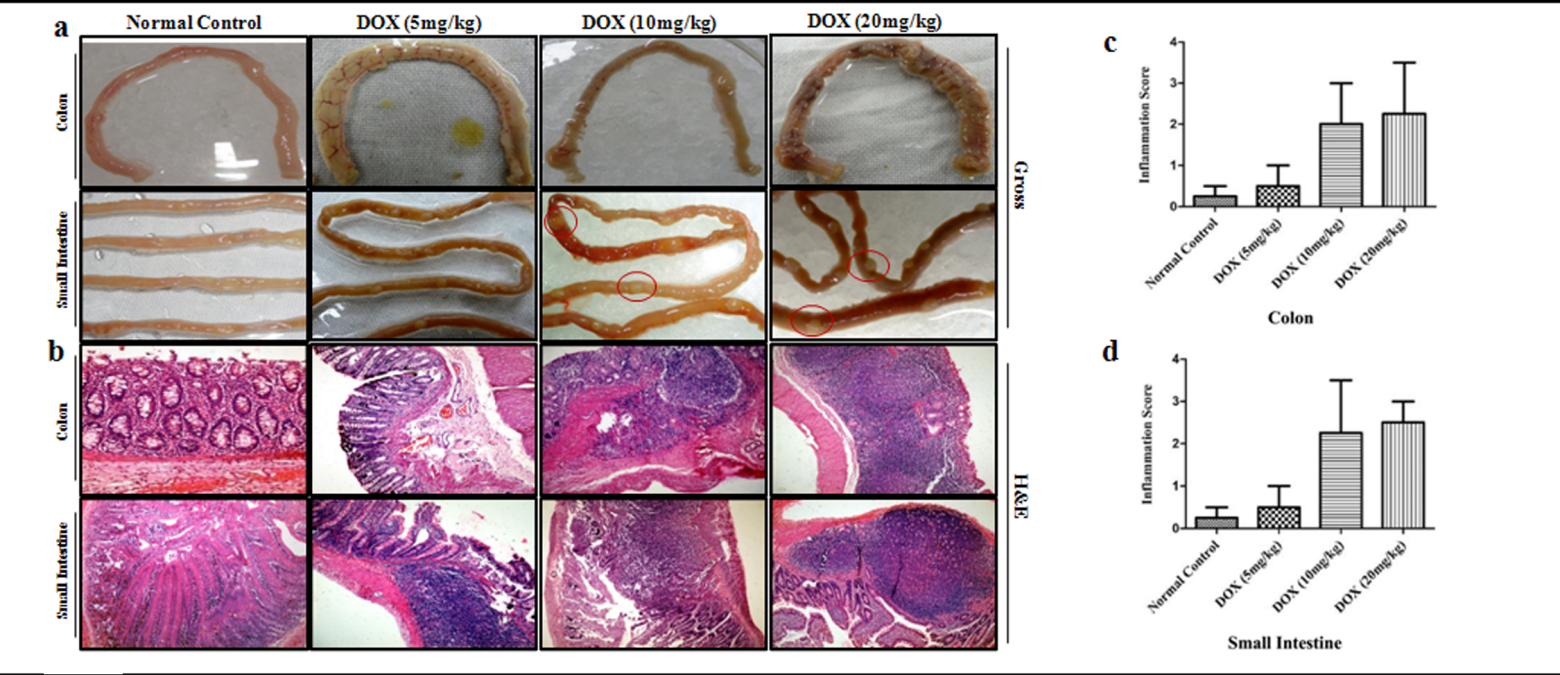
DOX stimulates chronic inflammation in colon and small intestine. **a)** Macroscopic examination of colon and small intestine of normal control, DOX-alone-treated rats **b)** Histopathological analysis of colon and small intestine of normal control and DOX-alone-treated rats; Hematoxylin and eosin staining (n = 24); Magnification ×100 **c,d)** Inflammatory scores of DOX-treated rats for chronic inflammation. Data are presented as Mean ± SD.

After observing an increase in primary tumor burden and metastatic spread by combined action of DMH and DOX, we studied the effect of DOX treatment alone on colon and small intestine in rats. For this purpose, doxycycline was administered in different doses (5,10,20 mg/kg bw) to a separate group of the animals. Macroscopically, DOX (5mg/kg) treated rats showed a marked increase in inflammation and vascular congestion of colon, whereas DOX (10mg/kg) and DOX (20mg/kg) treated rats showed colonic thickness, polypoid lesions and dullness of serosal aspect. Besides colon, all the rats treated with different doses of doxycycline developed numerous polyps on small intestine during the experimental period (Fig 2A). Photomicrographs of representative colonic sections demonstrated mild inflammation in DOX (5mg/kg) treated rats, whereas a dense inflammation in overlying mucosa with loss of crypts, reactive hyperplasia and mild dysplasia was observed in DOX (10mg/kg) and DOX (20mg/kg) treated rats. However, small intestinal sections of DOX (5mg/kg) treated rats showed inflammatory cell infiltration on mucosal layer, whereas DOX (10mg/kg) and DOX (20mg/kg) treated rats exhibited an expansion of peyer’s patches with chronic inflammation extending into the mucosa and sub-mucosa as well as increased mitotic figures, reactive hyperplasia and inflammatory atypia (Fig 2B). Inflammation scored on a 0–4 scale in colon and small intestine of all DOX treated rats. The results are shown in Table 4 and Fig 2C and 2D.

**Table 4:** Inflammation score of DOX treated rats.

	Inflammation Score
**Colon**	**(0–4 Scale)**
Control	0
DOX-5	0 (0–1)
DOX-10	3
DOX-20	3+ (3–4)
**Small Intestine**	**(0–4 Scale)**
Control	0
DOX-5	1 (0–1)
DOX-10	3 (2–3)
DOX-20	3+ (3–4)

### Doxycycline upregulates the expression of NF-κB, MMP-9 & VEGF, but downregulates p53, cytochrome-c, caspase-3 & caspase-9 expression

**Fig 3:**
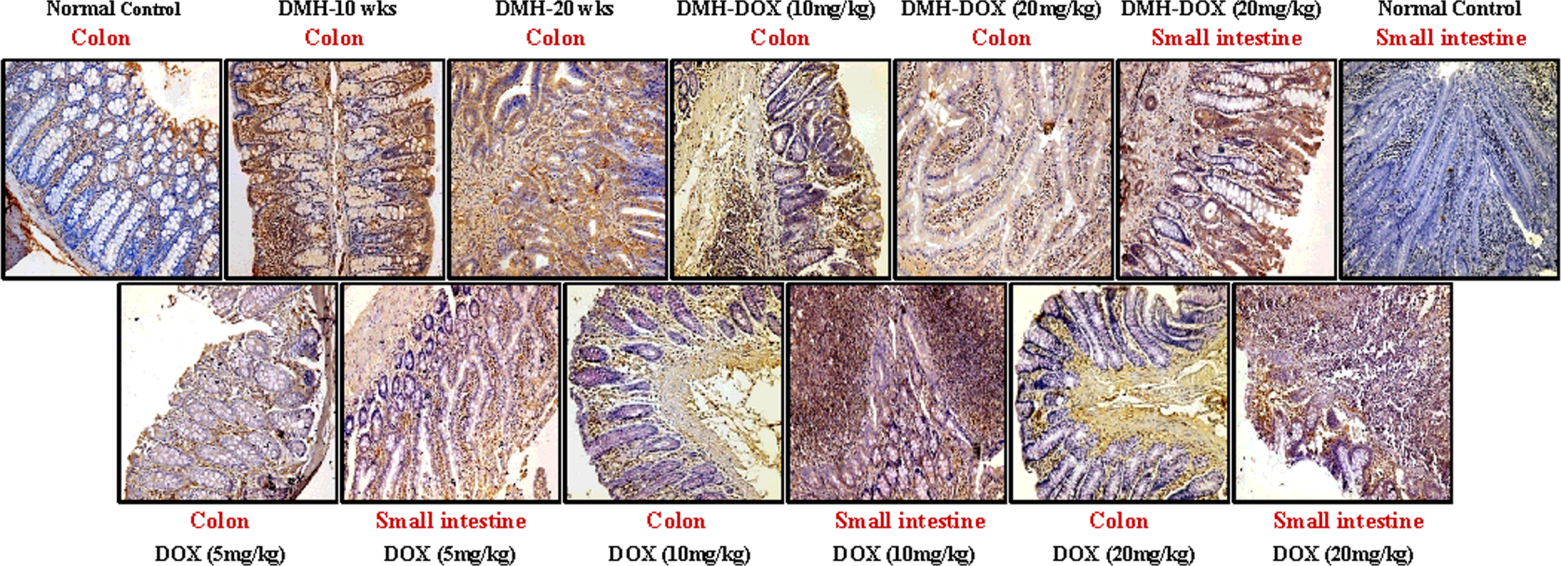
Immunohistochemistry for colonic and small intestinal tissues from normal control, DMH-alone, DMH-DOX and DOX-alone-treated rats. Immunostaining with anti-NF-κB. The signal for protein is represented by brown color due to DAB and blue signal due to hematoxylin counterstain. Magnification ×200; (n = 24).

**Fig 4:**
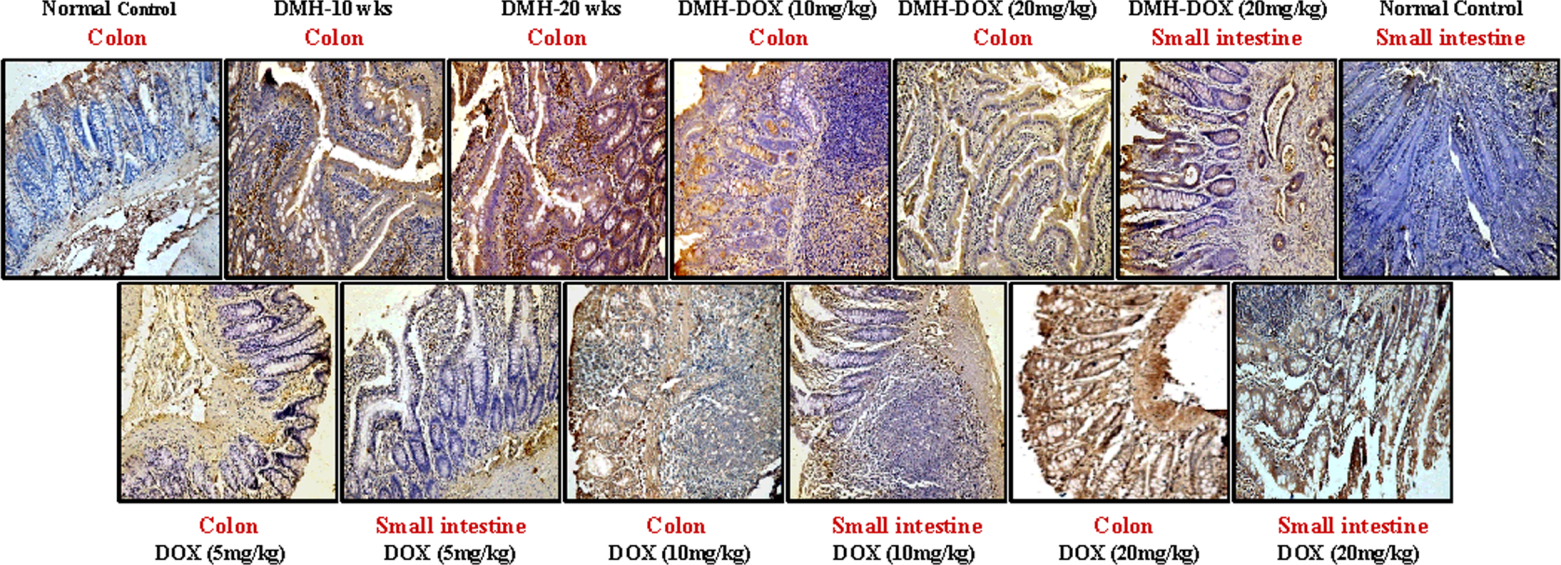
Immunohistochemistry for colonic and small intestinal tissues from normal control, DMH-alone, DMH-DOX and DOX-alone-treated rats. Immunostaining with anti-MMP-9. The signal for protein is represented by brown color due to DAB and blue signal due to hematoxylin counterstain. Magnification ×200; (n = 24).

**Fig 5:**
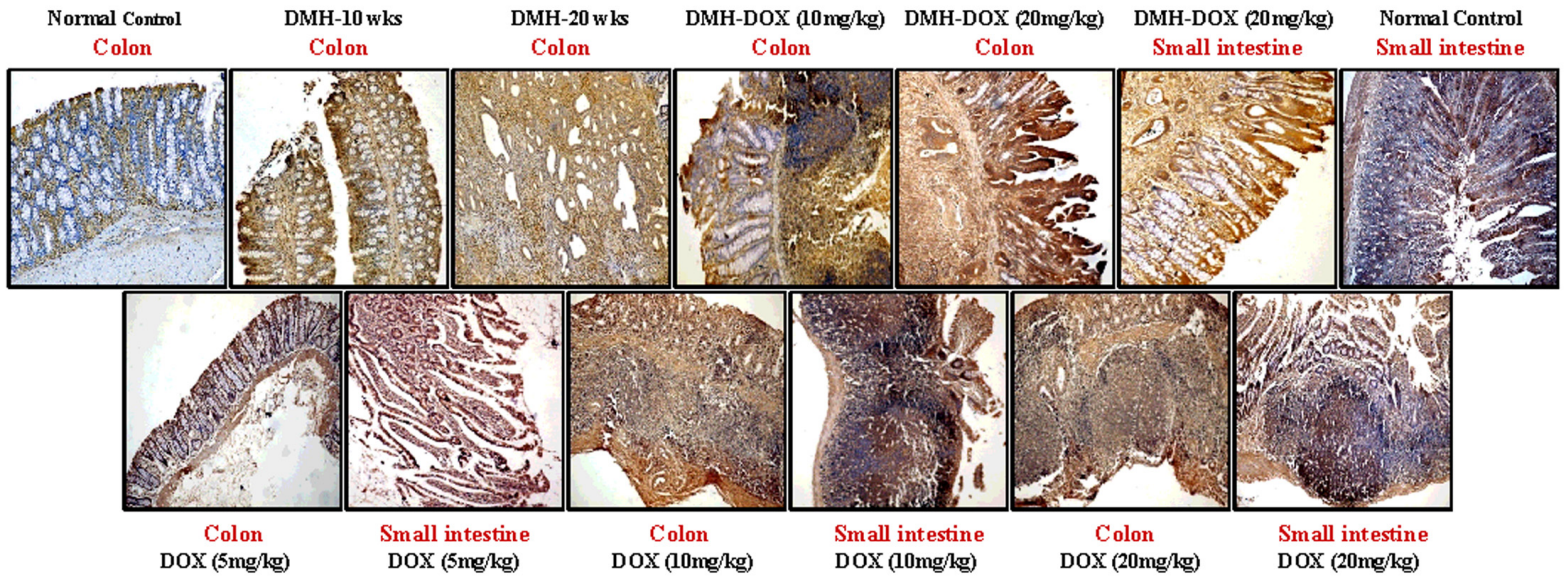
Immunohistochemistry for colonic and small intestinal tissues from normal control, DMH-alone, DMH-DOX and DOX-alone-treated rats. Immunostaining with anti-VEGF. The signal for protein is represented by brown color due to DAB and blue signal due to hematoxylin counterstain. Magnification ×200, ×100; (n = 24).

**Fig 6:**
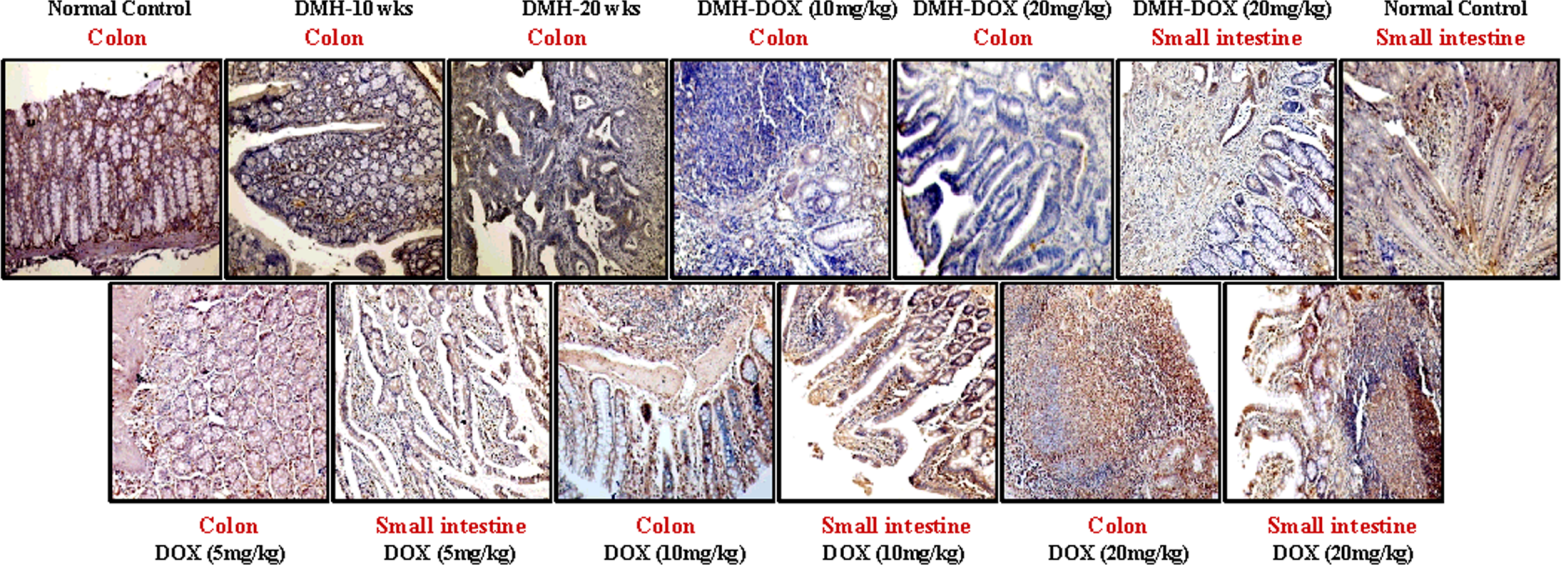
Immunohistochemistry for colonic and small intestinal tissues from normal control, DMH-alone, DMH-DOX and DOX-alone-treated rats. Immunostaining with anti-p53. The signal for protein is represented by brown color due to DAB and blue signal due to hematoxylin counterstain. Magnification ×200; (n = 24).

**Fig 7:**
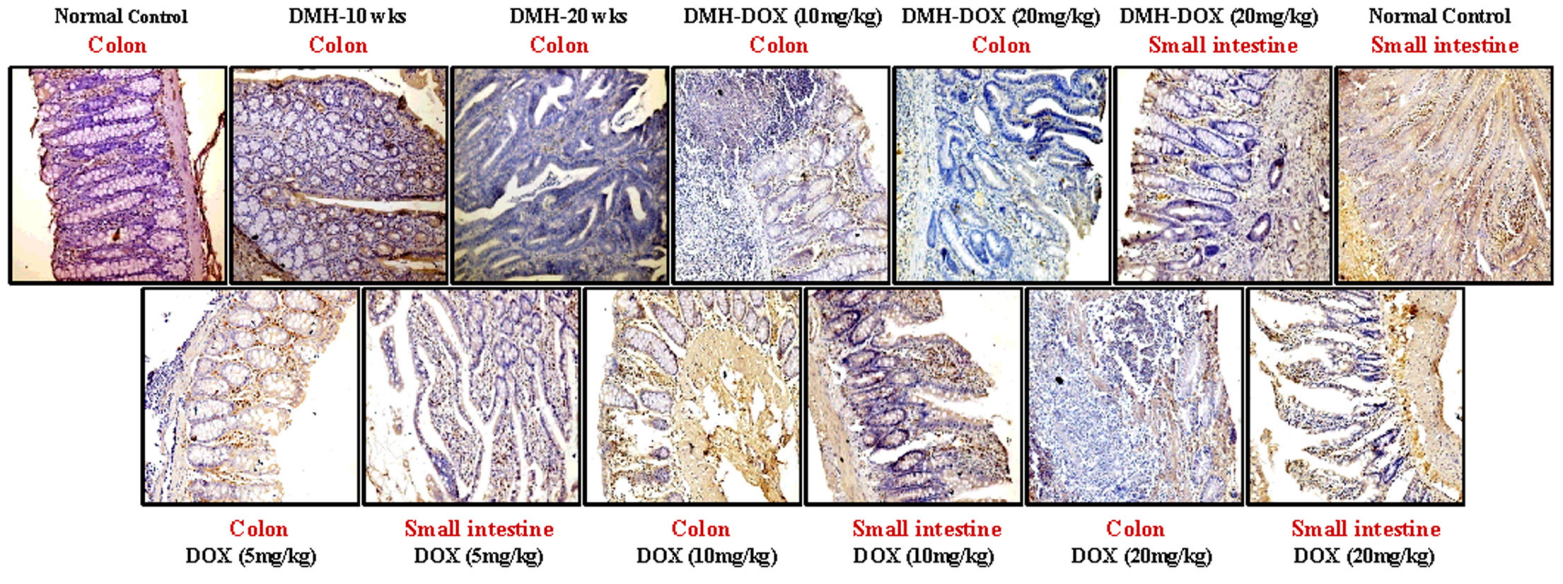
Immunohistochemistry for colonic and small intestinal tissues from normal control, DMH-alone, DMH-DOX and DOX-alone-treated rats. Immunostaining with anti-cyt-c. The signal for protein is represented by brown color due to DAB and blue signal due to hematoxylin counterstain. Magnification ×200; (n = 24).

**Fig 8:**
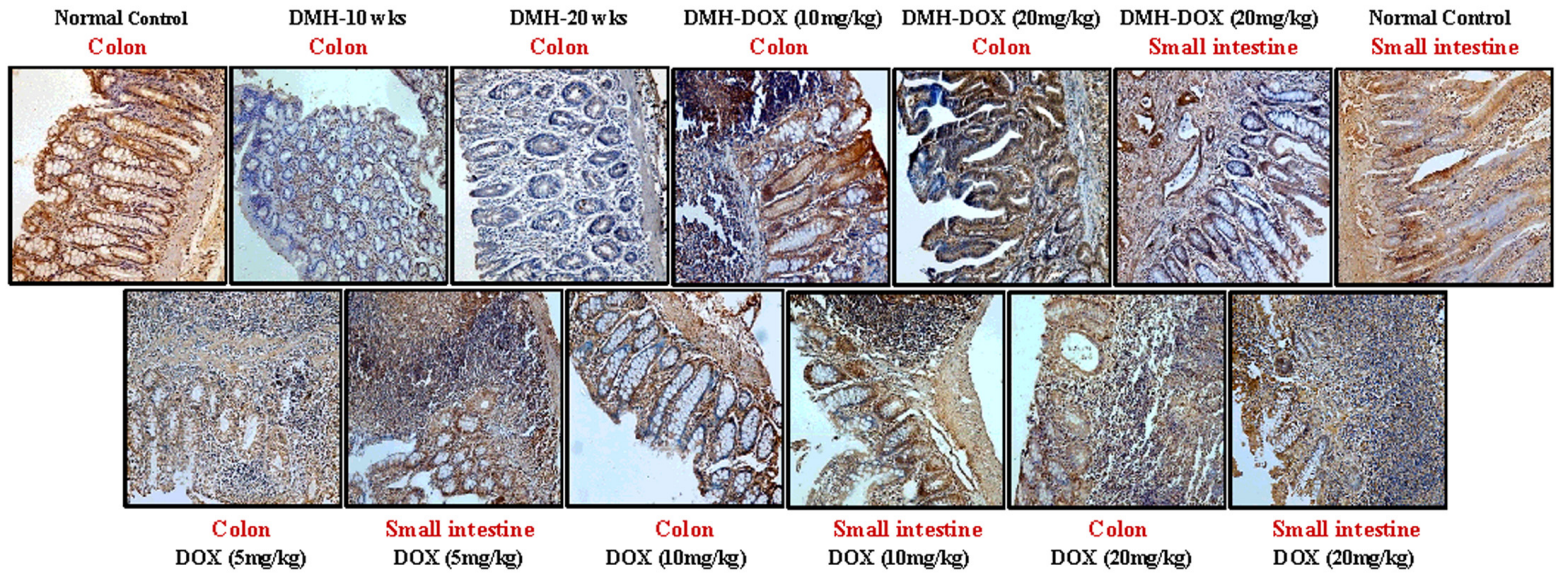
Immunohistochemistry for colonic and small intestinal tissues from normal control, DMH-alone, DMH-DOX and DOX-alone-treated rats. Immunostaining with anti-caspase-3. The signal for protein is represented by brown color due to DAB and blue signal due to hematoxylin counterstain. Magnification ×200; (n = 24).

**Fig 9:**
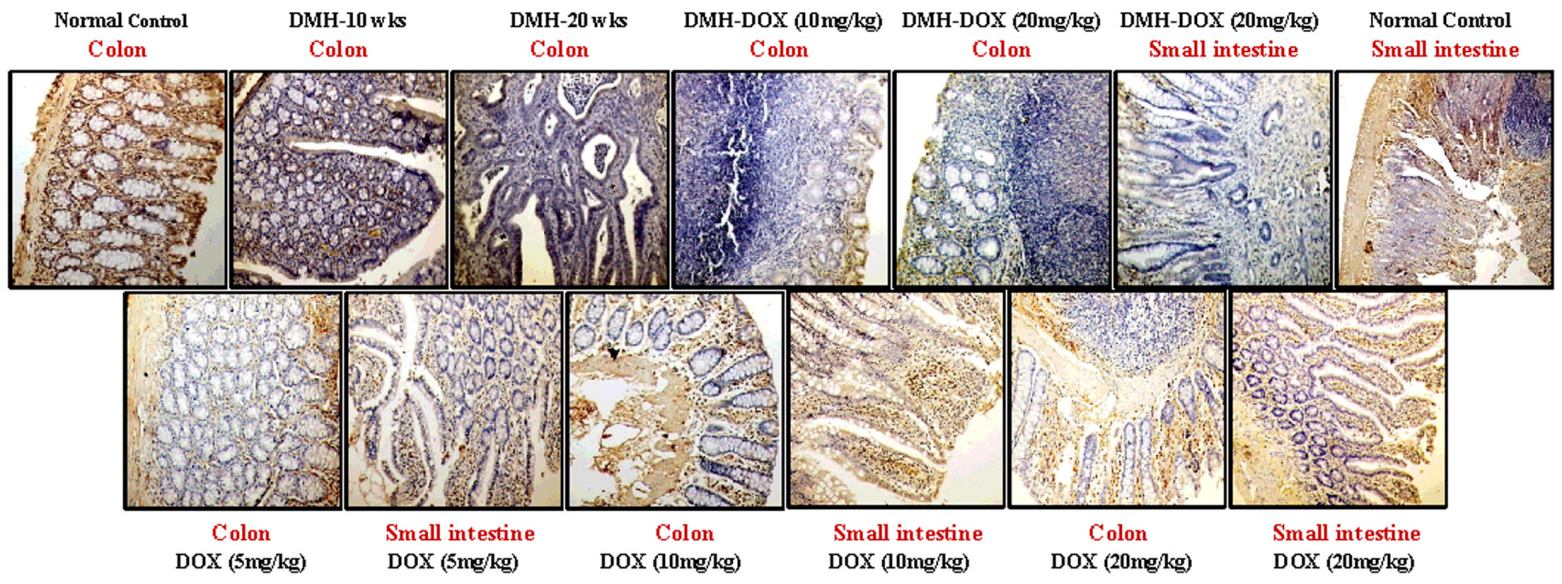
Immunohistochemistry for colonic and small intestinal tissues from normal control, DMH-alone, DMH-DOX and DOX-alone-treated rats. Immunostaining with anti-caspase-9. The signal for protein is represented by brown color due to DAB and blue signal due to hematoxylin counterstain. Magnification ×200; (n = 24).

The expression of NF-κB p65 protein in DMH-treated rats, DMH-DOX-treated rats and DOX-treated rats was augmented in comparison to normal rats. An increased NF-κB p65 protein expression was observed mainly in the nuclei of epithelial cells in treated animals, whereas, more cytoplasmic staining and weak nuclear staining was observed in normal animals (Fig 3). In addition, IHC analyses also indicated that relative to controls, treatment with DMH-alone and DMH-DOX resulted in the up-regulation of MMP-9 and VEGF expression, and a mild increase in DOX-treated rats (Figs 4 and 5). An augmented MMP-9 and VEGF expression was observed in the cytoplasm and membrane of epithelial cells in treated animals as compared to normal controls. Further, as shown in Fig 6, the expression of tumor suppressor protein p53 was predominantly nuclear, although some cells showed cytoplasmic expression in epithelium of normal or DOX-treated rats. However, an increase in cytoplasmic immunoreactivity and weak nuclear staining was observed in DMH-treated or DMH-DOX-treated rats. Apoptotic proteins viz. cytochrome-c, caspase-3 and caspase-9 were down-regulated in DMH-treated or DMH-DOX-treated rats as compared to DOX-treated or normal controls. The expression of these proteins was observed in cytoplasm only (Figs 7–9). Immunohistochemical staining of these proteins was scored on a 0–3 scale in all the groups as shown in Table 5.

**Table 5:** Immunohistochemical score of normal, DMH, DMH-DOX and DOX-treated rats.

	NF-κB (Nuclear)	MMP-9 (Cytoplasmic)	VEGF (Cytoplasmic)	p53 (Nuclear)	Cyt-c (Cytoplasmic)	Caspase-3 (Cytoplasmic)	Caspase-9 (Cytoplasmic)
**Normal Control (Colon)**	(1–2)+	1+	1+	3+	2+	2+	2+
**DMH-10 wks (Colon)**	3+	2+	2+	2+	(1–2)+	1+	(0–1)+
**DMH-20 wks (Colon)**	2+	2+	2+	(1–2)+	1+	(0–1)+	(0–1)+
**DMH-DOX (10mg/kg) (Colon)**	2+	2+	2+	1+	(0–1)+	(1–2)+	1+
**DMH-DOX (20mg/kg) (Colon)**	2+	2+	(2–3)+	1+	0	1+	(0–1)+
**DMH-DOX (20mg/kg) (Small intestine)**	3+	2+	2+	(1–2)+	1+	1+	0
**Normal Control (Small intestine)**	1+	0	(1–2)+	2+	2+	2+	2+
**DOX (5mg/kg) (Colon)**	2+	1+	2+	2+	1+	2+	1+
**DOX (5mg/kg) (Small intestine)**	2+	1+	2+	2+	1+	2+	1+
**DOX (10mg/kg) (Colon)**	2+	1+	2+	2+	(1–2)+	2+	1+
**DOX (10mg/kg) (Small intestine)**	3+	1+	2+	2+	1+	2+	1+
**DOX (20mg/kg) (Colon)**	2+	2+	2+	3+	1+	(1–2)+	(0–1)+
**DOX (20mg/kg) (Small intestine)**	2+	2+	(2–3)+	(2–3)+	1+	1+	1+

## Discussion

Doxycycline is the most widely used tetracycline that has been prescribed for years for various infections. Recently, several studies have shown its chemotherapeutic potency against several types of cancers, including colorectal cancer [7,8]. In the current study, we have developed an animal model by administration of known colon cancer promoter (DMH) for 10 and 20 weeks, resulted in the development of adenomas and adenocarcinomas respectively. DMH is a highly specific colon carcinogen, which is metabolized in the liver by a series of reactions through cytochrome P450 (P450) enzymes. Azomethane, AOM and methylazoxymethanol (MAM) are identified as the metabolites of DMH which are eventually converted into highly reactive methyldiazonium ion, that forms colonic DNA adducts and initiate the process of tumorigenesis [18,19].

Also, we observed the effects of DOX in DMH treated rats, so as to determine its effects on tumor regression in the early stages of colon carcinoma. But to our surprise, administration of DOX to DMH treated animals, promoted colon cancer progression followed by metastasis rather than tumor suppression. The present findings are in contrast to a couple of reports published in the literature, which described the anti-proliferative, cytotoxic and apoptotic nature of doxycycline [4–9]. Due to its antibiotic nature, doxycycline has been used in clinical medicine for several skin infections such as rashes, acne, abscesses, and lyme disease [3]. However, in the present study, unusual skin abscesses were observed at the injection site of DMH-DOX-treated rats which reflects the toxic effect of doxycycline. It was seen that treatment with DMH-DOX (10 mg/kg) and DMH-DOX (20 mg/kg) caused an increased in tumor multiplicity, tumor size and apparent shift from adenoma to carcinoma stage followed by metastasis (determined by histopathology). In contrast to these findings, Duivenvoorden et al. (2002) [5] demonstrated that doxycycline treatment decreased total tumor burden in bone metastasis model of human breast cancer with 70% reduction. The observed differences in the mode of action of DOX in the two studies may be due to different carcinoma models employed in these studies.

Further, the effect of different doses of DOX-alone on colon and small intestine revealed distinctly increased inflammation, benign polypoid lesions in colonic sections and numerous small benign lesions on small intestine. It is likely that doxycycline treatment can cause inflammation and lesions on other organs as reported by others [20, 21], however, to our knowledge, no study has reported the effect of doxycycline on colonic lesions under *in vivo* conditions.

The present results apparently demonstrated that administration of DMH followed by DOX caused additive or interactive effects by increasing either tumor multiplicity or tumor progression, thus suggesting that doxycycline enhances the effectiveness of DMH. Also, doxycycline treatment caused a more severe inflammatory response, which suggests that chronic inflammation becomes a major cofactor in the pathogenesis of cancer in the rats. The underlying reason for the contrary results in present study may be because of chronic inflammation induced by doxycycline which further would lead to enhanced efficacy of the carcinogen. Besides the above mentioned mechanism, the loss of commensal bacteria might be one of the possible mechanisms of doxycycline regulation, and can be explored by comparing with other non-tetracycline antibiotic. However, in the present study, our main focus was on the effect of doxycycline and its mechanism of action, so we did not compare its action with other non-tetracycline antibiotics but further studies are required to explore this point.

The protein expression of various biomarkers such as NF-κB, MMP-9, VEGF, p53, cyt-c, caspase-3 and caspase-9 related to colon carcinogenesis, indicated augmented expression of NF-κB in DMH-treated rats as well as DMH-DOX and DOX-treated rats compared to controls. These results are supported by earlier reports [22] which demonstrated that activation of nuclear factor kappa B (NF-κB), a proinflammatory transcription factor may have a promoting role in cancer development. It is evident that NF-κB gets activated through IL-1β/TNF dependent manner and its expression is dependent on the activation of NF-κB, which contributes to inflammation process [23, 24]. Activated NF-kB further regulates various inflammatory cytokines such as IL-1, IL-2, IL-6, IL-8, TNF and IFNγ that contribute to the pathogenesis of chronic inflammation and the development of CRC [22–25]. Moreover, NF- κB is known for its direct binding to other transcription factor such as STAT3 and its interaction promotes the development and progression of colon, gastric and liver cancers by regulating a number of cytokines and chemokines. It is also a well-known fact that STAT activation is generally mediated by members of the JAK family of tyrosine-kinases via stimulation of NF-κB regulated genes such as IL-6, IL-10 and IFNγ which are produced by tumor cells in an autocrine manner [26–29]. Similarly, Fujioka et al. (2004) [30] have shown that doxycycline strongly activates NF-κB through generation of reactive oxygen species (ROS) in human pancreatic tumor cell line. Earlier, reports suggested that ROS generation is triggered by activation of tumour necrosis factor receptor (TNFR), which stimulates the phosphorylation of IκB, a NF-κB inhibitor via oxidative inhibition of a phosphatase and leads to the proteasomal degradation of IκB, thereby resulting in the nuclear translocation of NF-κB [31, 32]. Recently, Wang et al. (2015) [33] indicated that ROS mediated NF-κB activation promotes tumor growth and metastasis by up-regulating NF-κB target genes (VEGF and MCP-1) in hepatocellular carcinoma.

Though, the levels of TNF and ROS were not investigated in the present study, but the possible mechanism for NF-κB activation and increased expression by DMH and DOX is presumably due to ROS generation or via TNF-α-induced IKK-β-dependent NF-κB-activation pathway in rats.

MMPs are involved in cancer biology for over 40 years, and it is believed that MMPs promote the degradation of extracellular matrix (ECM) that leads to the cancer cell invasion and metastasis [34]. MMP-9 plays an important role in tumor angiogenesis by modulating the bioavailability of vascular endothelial growth factor (VEGF), a tumor angiogenesis marker and a promising therapeutic target [35]. Several studies have shown the MMP-9 inhibition by doxycycline, so, before analyzing the expression of MMP-9 in the current study, we assumed that MMP-9 would be inhibited or reduced by doxycycline which could lead to the increased angiogenesis and tumor growth. The ground behind these assumptions were paradoxical effect of MMP-9 which demonstrated that reduction of plasma levels of MMP-9 leads to decreased angiostatin synthesis and subsequent increased tumor growth and vascularization [36,37]. However, we found increased expression of MMP-9 in DMH or DMH-DOX-treated, and mild up-regulation in DOX-treated rats. These findings are contradictory to those reported by Dursun et al. (2001) [38], suggesting that an anti-tumorigenic role of doxycycline by inhibition of MMP-9. Akkaya et al. (2009) [39] have reported regression of endometeriosis in rats by DOX induced MMPs inhibition, Onoda et al. (2004) [8] also described an anti-invasive effects of DOX with attenuated MMP expression and its activity in colon cancer cell line. The possible reason underlying these contradictory results may be the NF-κB dependent regulation of metastatic pathway by activation of MMPs, resulted in NF-κB mediated metastasis as reported by Nakanishi et al. (2005) [40].

Further, up-regulation of VEGF in DMH or DMH-DOX-treated rats, and slight increase in DOX-treated animals was observed. Angiogenesis is essential for tumor progression and is dependent upon several signaling pathways, with greater importance of the VEGF (vascular endothelial growth factor) pathway [41]. Although an increased expression of VEGF in doxycycline treated group was observed, but some studies have reported that doxycycline prevents VEGF-induced vascular permeability, and inhibit angiogenesis in both humans and animal models [42]. The underlying basis of these contradictory findings might be the augmentation of MMP-9 activity, as shown by Bergers et al. (2000) [35]. It is also likely that VEGF and MMPs were up-regulated by NF-κB-dependent manner, resulting in tumor angiogenesis and metastasis [43].

The down-regulation of p53 expression in DMH or DMH-DOX-treated rats as compared to DOX-treated or control rats was also observed. The tumor suppressor protein p53 (TP53) regulates cell cycle, genomic stability, apoptosis, inhibits angiogenesis and is mutated at high frequency in human cancers [44]. Recently, our group has reported p53 mutations in CRC samples from human subjects, which suggests the poor survival of patients in Indian population [45]. Common mutant p53 (mutp53) directly enhance cancer progression and development through gain-of-function (GOF) mechanisms [44,46]. Also, mutp53 has been reported to induce constitutive NF-κB activation by TNF-α, while activated NF-κB inhibits p53-dependent transactivation. This is consistent with a mutual repression mechanism and could contribute to chronic inflammation and inflammation-associated colorectal cancer [46,47].

Doxycycline has cytotoxic actions and apoptotic effects in multiple carcinomas [4–6]. In colon cancer cell lines, doxycycline increases the level of cytosolic cytochrome-c and triggers caspase-3 and caspase-9 activity which in turns leads to the caspase-dependent apoptosis [6]. For this, we investigated the apoptotic activity of doxycycline, but observed reduced expression of cytochrome-c, caspase-3 and caspase-9 in DMH-alone and DMH-DOX-treated rats as compared to DOX-treated and control rats which shown the anti-apoptotic activity of doxycycline. The probable factor behind these findings might be the NF-κB promoted cell survival through expression of anti-apoptotic genes that block the caspase cascade [48] or anti-apoptotic members of the Bcl family that act at the mitochondrial level and inhibit cytochrome c release which could be involved in apoptosis [49].

To date, several *in vitro* studies have reported the apoptotic nature of doxycycline in various cancers including colon cancer, but none of the study could be validated under *in vivo* conditions of colon cancer. The present study demonstrated the anti-apoptotic activity of doxycycline under *in vivo* conditions. Earlier, two *in vivo* studies in breast cancer reported the anti-tumor effects of doxycycline by suppression of MMPs activity and inhibition of tumor growth. Duivenvoorden. (2002) [50] reported the attenuated MMPs activity in breast cancer by administration of doxycycline in time release pellets, and demonstrates its benefit when administered from the time of the development of the tumor. In another study, [51] athymic mouse xenograft model of breast cancer was used which received doxycycline in drinking water and inhibits gelatinolytic activity and cell proliferation. However, in the current study, we injected doxycycline intraperitoneally after the development of adenoma in animal model of colon carcinoma and analysed its invasive activity by observation of augmented MMP-9 levels. Being a contrary finding from earlier reports, we have repeated our study to confirm these findings and the results were consistent as before.

In conclusion, present findings suggest that doxycycline treatment may cause chronic inflammation in the rat colon, small intestinal injury and enhances effectiveness of DMH by causing multiple tumors at an early stage (adenoma) of colon carcinogenesis. Thus provides evidences for a connection between doxycycline induced chronic inflammation and cancer. Nevertheless, NF-κB is a key regulator of inflammation, which may precede tumor initiation process followed by tumor promotion and progression [22], is implicated in this phenomenon.

**Fig 10:**
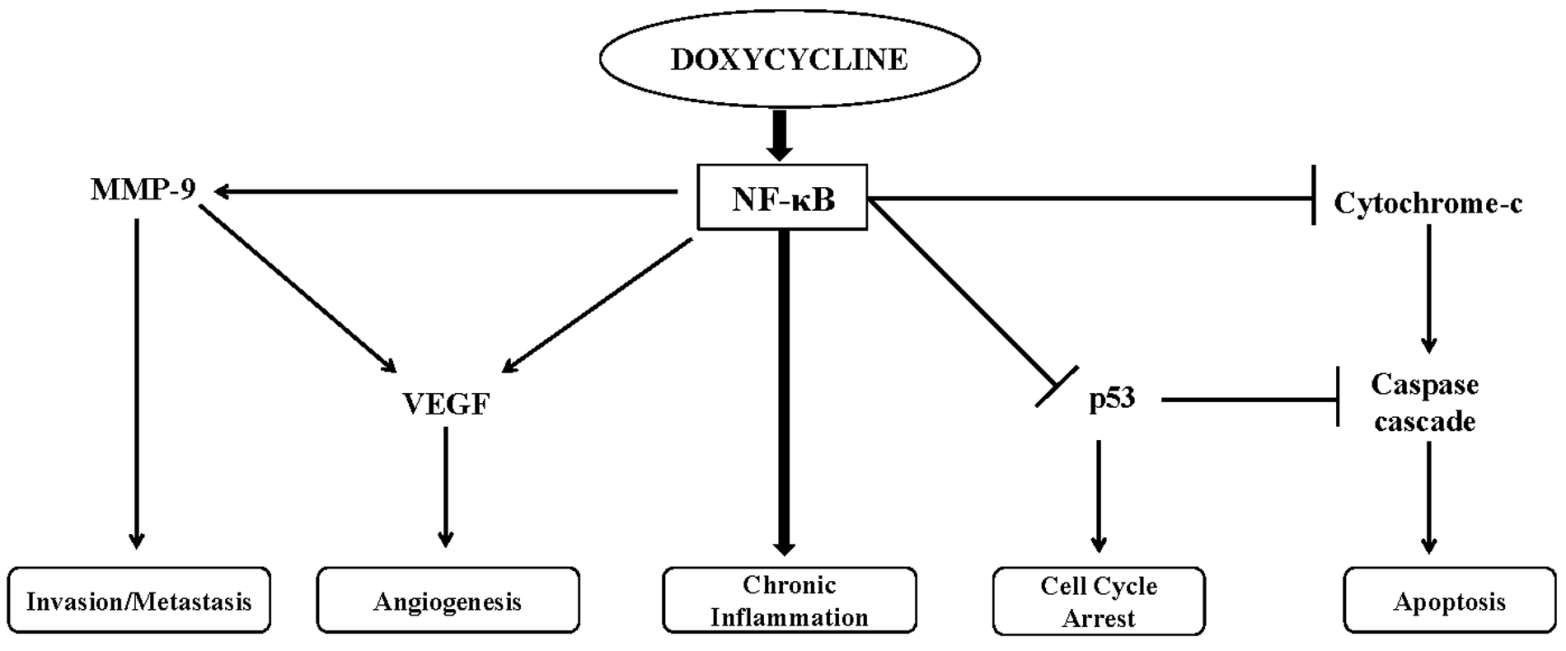
Hypothetical model demonstrates the role of doxycycline induced chronic inflammation drive carcinogénesis by activation of NF-κB pathway.

We propose that doxycycline causes chronic inflammation by activation of NF-κB, which further regulates MMP-9, VEGF, p53, cytochrome c and caspases (Fig 10). Although we demonstrated translational expression of above biomarkers, but further studies are required to explore molecular mechanism(s) of doxycycline-induced inflammatory process resulting in carcinogenesis, and understanding the function of NF-κB in context of inflammation induced tumorigenesis.
